# Metabarcoding analysis on European coastal samples reveals new molecular metazoan diversity

**DOI:** 10.1038/s41598-018-27509-8

**Published:** 2018-06-14

**Authors:** David López-Escardó, Jordi Paps, Colomban de Vargas, Ramon Massana, Iñaki Ruiz-Trillo, Javier del Campo

**Affiliations:** 1 Institut de Biologia Evolutiva (CSIC-Universitat Pompeu Fabra), 08003 Barcelona, Catalonia Spain; 2 School of Biological Sciences, University of Essex, Wivenhoe Park, Colchester, CO4 3SQ UK; 3 CNRS, UMR 7144, Adaptation et Diversité en Milieu Marin, Station Biologique de Roscoff, Roscoff, France; 4 UPMC Univ. Paris 06, UMR 7144, Station Biologique de Roscoff, Roscoff, France; 5 Department of Marine Biology and Oceanography, Institut de Ciències del Mar (CSIC), Barcelona, Catalonia Spain; 6 ICREA, Pg. Lluís Companys 23, 08010 Barcelona, Catalonia Spain; 7 Departament de Genètica, Microbiología i Estadística, Universitat de Barcelona, Barcelona, Catalonia Spain

## Abstract

Although animals are among the best studied organisms, we still lack a full description of their diversity, especially for microscopic taxa. This is partly due to the time-consuming and costly nature of surveying animal diversity through morphological and molecular studies of individual taxa. A powerful alternative is the use of high-throughput environmental sequencing, providing molecular data from all organisms sampled. We here address the unknown diversity of animal phyla in marine environments using an extensive dataset designed to assess eukaryotic ribosomal diversity among European coastal locations. A multi-phylum assessment of marine animal diversity that includes water column and sediments, oxic and anoxic environments, and both DNA and RNA templates, revealed a high percentage of novel 18S rRNA sequences in most phyla, suggesting that marine environments have not yet been fully sampled at a molecular level. This novelty is especially high among Platyhelminthes, Acoelomorpha, and Nematoda, which are well studied from a morphological perspective and abundant in benthic environments. We also identified, based on molecular data, a potentially novel group of widespread tunicates. Moreover, we recovered a high number of reads for Ctenophora and Cnidaria in the smaller fractions suggesting their gametes might play a greater ecological role than previously suspected.

## Introduction

The animal kingdom is one of the best-studied branches of the tree of life^1^, with more than 1.5 million species described in around 35 different phyla^2^. Some authors have suggested there may be more than 10 million species of animals, indicating that there is an extensive unknown animal diversity. This hidden diversity may vary according to the animal phyla considered. Not surprisingly, those animal phyla with microscopic representatives (i.e., those animals with a size below 2 mm^3^, also known as micrometazoans^4^) are suggested to contain most of this potential unknown diversity^3^.

Marine environments cover most of the earth’s surface. More importantly, all metazoan phyla, except onycophorans, have marine representatives, with up to 60% including microscopic members^5^. Copepods, for instance, are the most abundant multicellular group of organisms on earth^6^, highlighting the key role of microbial animals in marine ecosystems. Given that the marine benthic meiofauna is also one of the hot spots of alpha-diversity in the biosphere, marine environments thus appear to be ideal sites in which to analyze animal diversity across phyla.

Classical methods to survey animal diversity, such as isolation and morphological identification, might be ineffective to comprehensively analyze micro/mesozooplanktonic^7^ and meiofaunal diversity^8^. The microscopic size of the organisms and the wide variety of morphologies makes the identification process tedious and slow, requiring taxonomists with experience in different groups to properly assess the composition of the community and describe new species or groups. Molecular techniques, and especially high-throughput environmental sequencing (HTES), have recently provided a more efficient method to assess and understand ecological patterns in the microbial world^9^, including metazoans^8,10–12^. However, these studies have mainly focused on richness patterns in marine benthic communities or in zooplanktonic communities, with special attention on copepods^7,13^. Studies of microbial eukaryotes^14–16^ and even some animal clades^17^ suggest that HTES could also be used to detect novel lineages. However, such an approach has yet to be applied across the whole animal kingdom.

**Figure 1:**
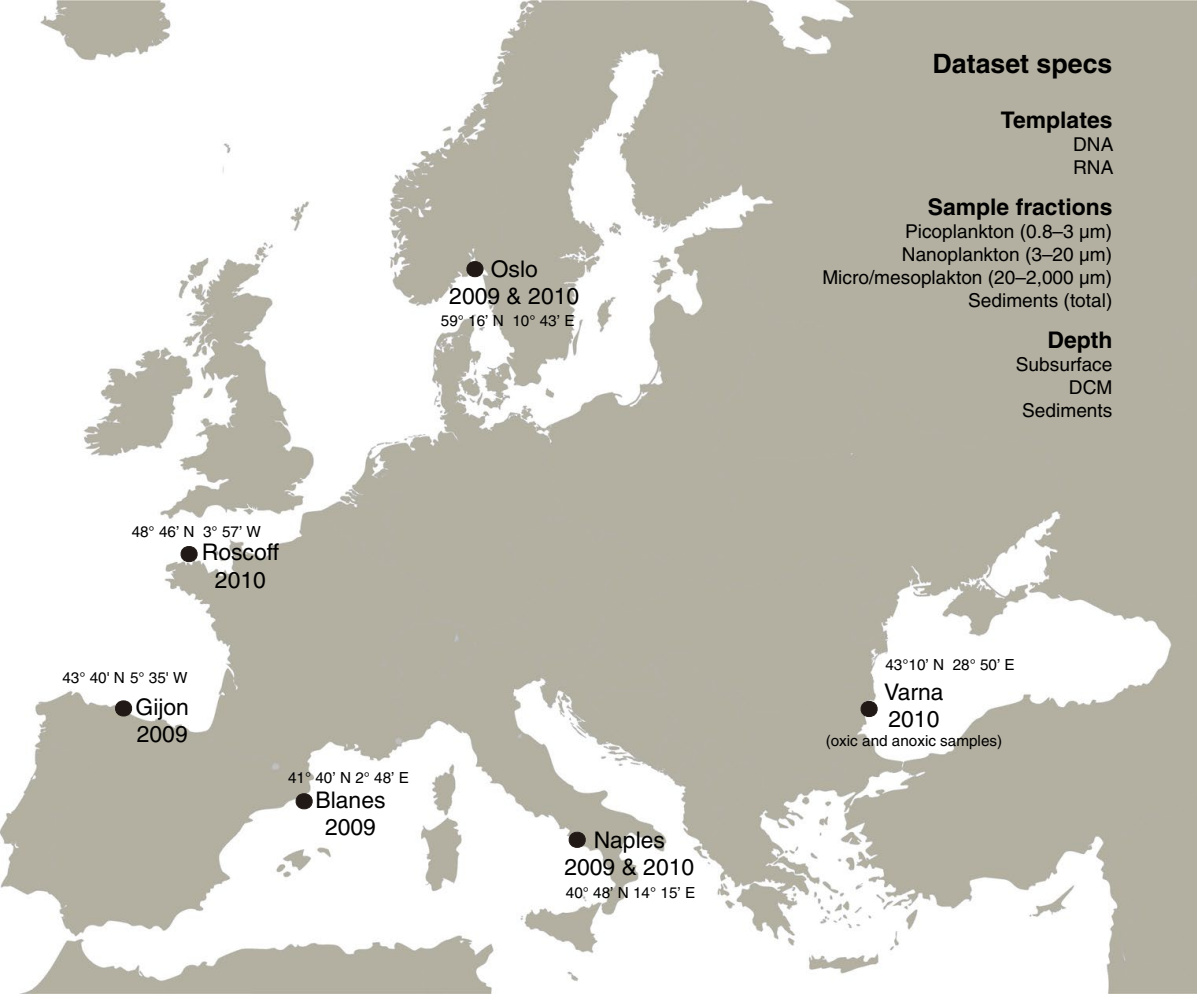
BioMarKs sampling sites. Map indicating the sampling locations where the data were collected and the summary of the dataset characteristics. Map retrieved from Wikimedia Commons (https://commons.wikimedia.org/wiki/File:Blank_map_Europe_without_borders.png) CC-BY-SA-4.0,3.0,2.5,2.0,1.0 (https://creativecommons.org/licenses/by-sa/4.0/).

To obtain a better understanding of the genetic diversity of the different metazoan phyla, and the potential of HTES to quantify diversity and novelty levels, we analyzed a large dataset of ribosomal small subunit (18S rRNA) V4 region tags from European coastal sampling sites in the context of the BioMarKs project, which was designed to analyze the diversity of unicellular eukaryotes. The BioMarKs dataset is based on 137 RNA and DNA samples from six locations^14,18^ (Fig. 1; Table S1). The use of RNA in this dataset allows analysis that goes beyond the detection of cells or DNA material in the environment, as it provides a window on biological activity due to the rapid RNA deterioration after cell death^19^. Previous studies using both RNA and DNA data, showed that DNA mostly recovers more number of OTUs^20^ which may belong to artifacts or rest of organisms^21^, while RNA data correlates better with alpha-diversity patterns^22^. Thus, thanks to the information from both templates we can have a more complete and reliable vision of our samples. In addition, for each sampling site, there is data from both pelagic and benthic environments, with the pelagic samples being divided into different depths and size fractions (Table S2).

The large quantity of data, together with the use of a phylogenetically curated taxonomic assignment has provided a global view of genetic diversity across all metazoan phyla. Our data show that 18S rRNA HTES approaches can be used to infer diversity and novelty. Furthermore, we provide evidence that many unsampled lineages remain among animals, and that there are even some potential novel groups. Consequently, greater efforts should be made to sample specific animal groups, especially in benthic environments.

## Results

### Metazoan18S rRNA reference database

An important point to consider when analyzing diversity by metabarcoding is how the taxonomic assignment is done. It is known that the use of GenBank or SILVA as reference databases to perform the taxonomic assignment^7,8,12,13,23,24^ can be problematic^25^. The reasons are different in both cases, while GenBank may contain numerous missannotations that affect the final taxonomic assignment; SILVA, despite being partially curated and checked via phylogenetic placement, is sometimes too strict in the filtering step, which may lead to a loss of diversity. In addition, SILVA taxonomical ranks are not updated in some eukaryotic lineages^26^, including animal taxa. Thus, to avoid these problems and to have the best possible taxonomic assignments, we manually constructed a novel phylogenetically curated metazoan 18S rRNA reference dataset.

Our database included 19,364 18S rRNA sequences retrieved from GenBank. The database was curated in a phylogenetic-wise manner, so that each animal phylum had the widest possible representation of internal groups and that each sequence had a clear taxonomic assignment. The resulting database was subsequently used to assign a taxonomic identity to the approximately 1.5 million reads analyzed, providing a holistic and phylogenetically accurate view of the metazoan diversity.

### General abundance and richness patterns of microbial animals

**Figure 2:**
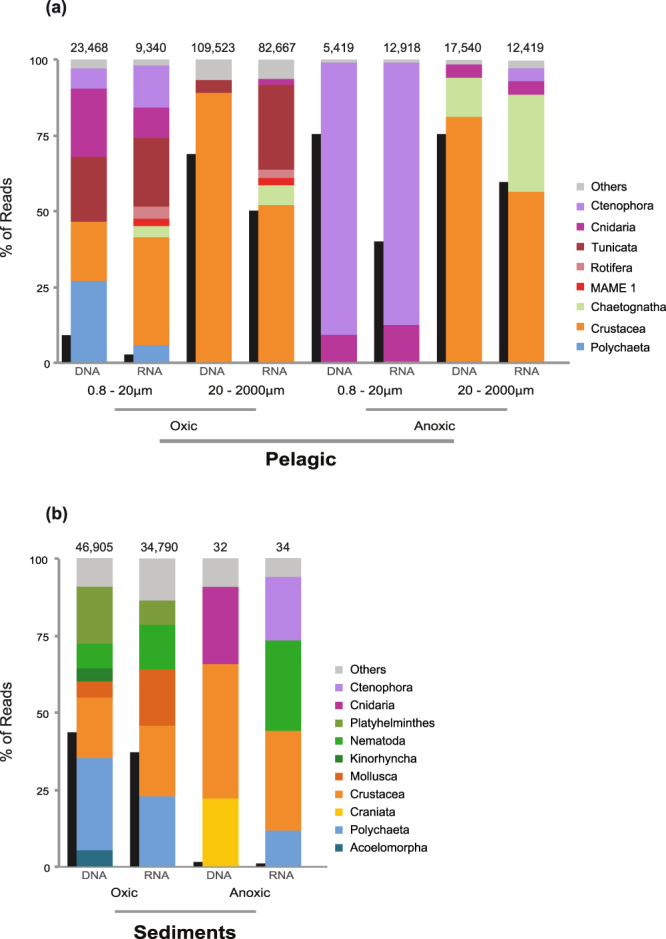
Relative abundances of different metazoan groups and metazoan relative abundance compared to the eukaryotes. Relative abundances of different metazoan groups (colored columns) and metazoan relative abundance compared to total eukaryotes (black columns) in (**a**) pelagic and (**b**) benthic samples (sediments) separated by oxygen availability and by template (source DNA or RNA). Pelagic data is divided also by size fraction. The number above each column represents the total number of metazoan reads present dataset.

We first analyzed the relative abundance of metazoan reads within the whole eukaryotic dataset. We found that metazoans reads were quite abundant compared to other eukaryotic groups in both the DNA and RNA samples (Figs 2; S1). This high percentage of metazoan reads was especially notable in anoxic pelagic environments (Fig. 2a) and in oxic sediments (Fig. 2b). Interestingly, metazoan reads were not only abundant in the micro/mesoplankton fraction (68% DNA, 49% RNA of the total eukaryotic reads), but also in the smaller fractions (i.e., the pico/nano fractions which are less than 20 um). The presence of a high percentage of metazoan reads in the smaller fractions is especially relevant in the anoxic environment, with 75% of the DNA reads (and 33% of the RNA) being assigned to metazoans.

**Figure 3:**
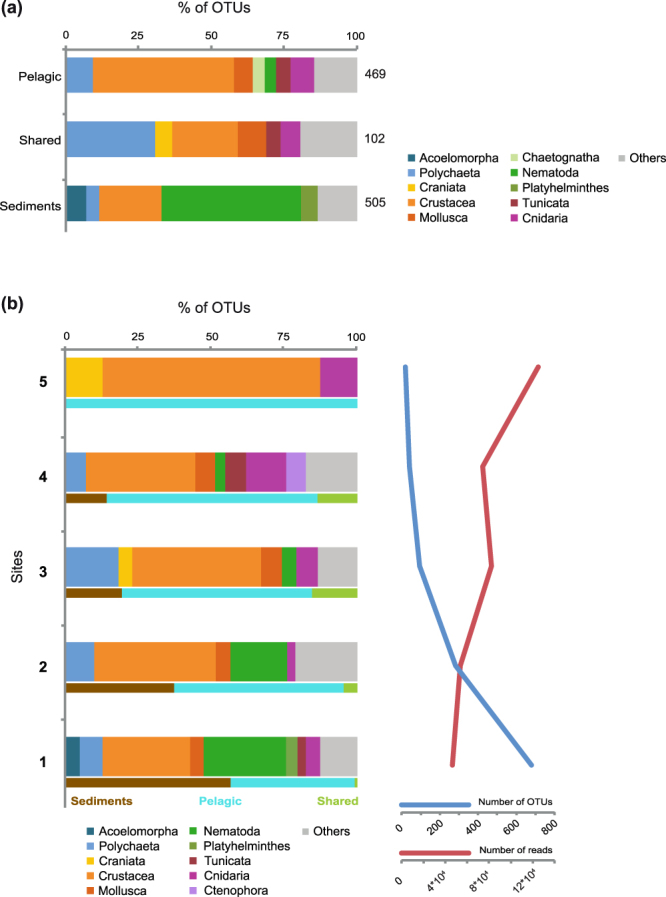
Metazoan richness. (**a**) OTU distribution for each metazoan group divided into pelagic specific, sediment specific and those present in both environments. The number on the right shows the total number of OTUs in each dataset. (**b**) Taxonomical distribution of the OTUs classified according to their occurrence in 1 or more (up to 5) geographical sites. It is also included the environmental distribution of OTUs based on prevalence: In blue, pelagic-specific OTUs (i.e., OTU with more than 90% of the reads within the water column); in green, OTUs present both in the water column and the sediments; in brown, OTUs present only in sediments (i.e., OTUs with more than 90% of the reads within the sediments). The number of OTUs (blue line) and number of reads (red line) based on their occurrence in 1 or more (up to 5) geographical site is shown to the right.

The clustering of reads into OTUs yielded 1067 OTUs from 23 different metazoan phyla (Fig. 3, Table S4). The rarefaction curves show that using RNA and DNA templates has allowed increasing the diversity recovered compared to using just one of the templates (Fig. S2). Actually, 80% of our OTUs have reads either from RNA or DNA data. Regarding the environmental distribution, 469 OTUs were found to be exclusive to the benthos, 505 to pelagic environments and 102 OTUs were present in both (Fig. 3a). Crustacea appeared as the richest clade (246 OTUs) within the pelagic-exclusive dataset, followed by Polychaeta (45). Within the benthic (sediment)-specific samples, the largest number of OTUs were from Nematoda (227), followed by Crustacea (101). Polychaeta (31) and Crustacea (23) dominated the OTUs present in both environments (Fig. 3a).

The largest proportion of animal reads in oxic water column environments were from Crustacea, which represented up to the 89% of DNA and 53% of RNA in the overall metazoans reads from the micro/meso fractions (Fig. 2a). More than 80% of the crustacean RNA reads, however, corresponded to 8 specific OTUs that were assigned to copepods (Table S5). Besides crustaceans, there was also a high abundance of reads from tunicates (5% DNA only, but 28% RNA) within the oxic micro/mesoplanktonic samples, most of them corresponding to appendicularians (Table S5). On the other hand, benthic samples were dominated by polychaetes (30% DNA, 23% RNA) and crustaceans (19% DNA, 23% RNA) (Fig. 2b). Within benthic Crustacea, ostracods and copepods were the most abundant groups (Table S6).

### Community structure across environments and size fractions

To determine the biogeographical patterns of the microbial animals in our dataset, we analyzed the presence/absence of OTUs in all five sites (discarding the anoxic samples). A large fraction of the OTUs (668 out of 1076) were present in just one single location. However, the number of reads of these “endemic” OTUs (around 4·10^4^) was three times lower than the 8 OTUs present in all sampling sites (around 1.2·10^5^ reads) (Fig. 3b). The taxonomic composition of the cosmopolitan OTUs (Fig. 3b) differed greatly from the complete dataset except for the crustacean dominance (Fig. 3b). In particular, there were no nematodes or polychaetes among the cosmopolitan OTUs, whereas a cnidarian and a craniate OTU appeared to be present over the 5 sampling sites. Our analysis also showed that all the cosmopolitan OTUs belonged to the water column, whereas more than half (56%) of the “endemic” ones belonged to the sediments. These endemic OTUs represented 80% of the total benthic OTUs.

On the other hand, we found a relatively high percentage of RNA reads in pelagic samples assigned to metazoans in the smaller fractions (from 0.8 to 20 µm): 2.4% in oxic and 32.4% in anoxic samples (Fig. 2a). As RNA reads indicate metabolically active cells^27^, we decided to analyze the potential source of those RNA reads. Most of the reads were crustaceans (36% RNA reads), followed by tunicates, ctenophores, cnidarians and polychaetes (Fig. 2a). Ctenophores (85% RNA pico/nano fractions) and cnidarians (16% RNA pico/nano fractions) dominated the reads assigned to metazoans in the anoxic waters of Varna, Black Sea (Fig. 2a).

**Figure 4:**
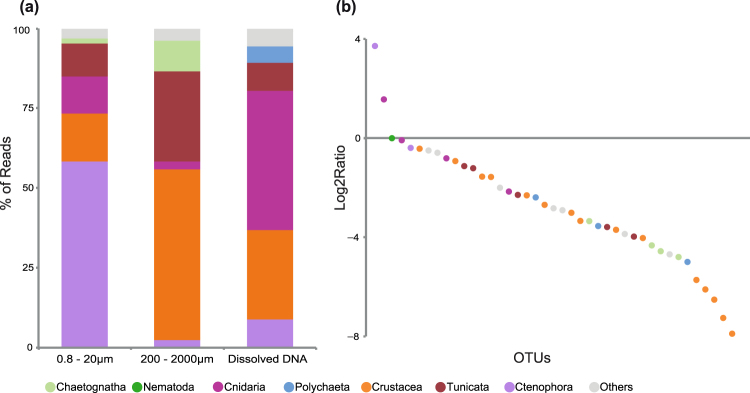
Analysis of the small (pico and nano) and large (micro/meso) fractions, and extracellular DNA. (**a**) Taxonomic distribution of the OTU reads in the smaller and larger fractions and within the extracellular DNA. (**b**) Ratio of the numbers of reads from the smaller fractions and large fraction for these OTUs.

To understand whether the reads from the smaller fractions were directly derived from the larger ones, we filtered the data based on their co-occurrence between the pico/nano fraction and the micro/meso fractions. We observed that OTUs present in both smaller and larger fractions had a clearly different proportion of reads (Fig. 4a). Most of the reads in the smaller fractions belonged to the ctenophores (58%), whereas crustaceans dominated (52%) the micro/mesoplanktonic fractions. In this regard, OTUs corresponding to *Pleurobrachia pileus* (a ctenophore) and *Aurelia aurita* (a cnidarian) were especially enriched in the smaller fraction (Fig. 4b), representing 57% of all metazoan RNA reads, and up to 33% of all eukaryotic RNA reads in the anoxic samples (Table S7) (Fig. 2a).

### Sequence novelty

**Figure 5:**
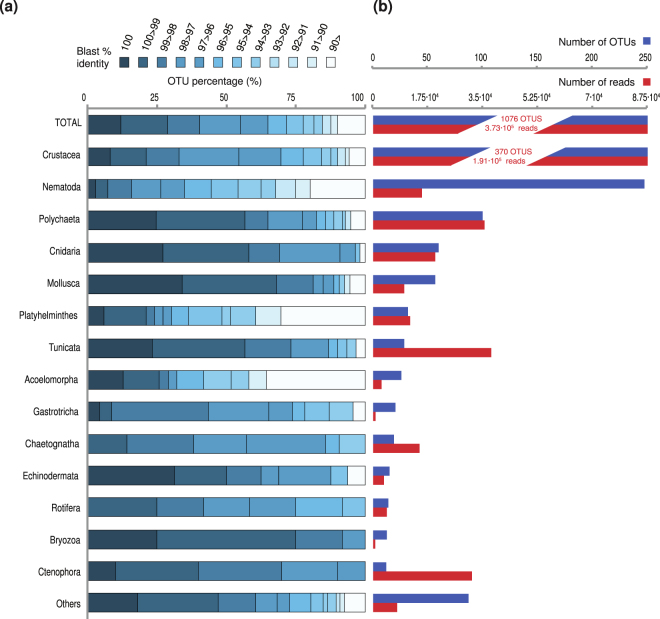
Sequence novelty plus summary of OTUs/read numbers of the main metazoan phyla in our dataset. (**a**) Distribution of OTU BLAST identities against NCBI nt nr for the main phyla of our dataset. (**b**) Summary of the number of OTUs (blue) and the number of reads (red) of the given phyla.

We performed BLAST searches against the NCBI nt nr database to interrogate the level of novelty in our molecular dataset across all animal phyla. The results revealed a high degree of sequence novelty (Fig. 5). In particular, 35.5% of our OTUs (representing 10.5% of the reads) had a BLAST identity lower than 97% compared to NCBI sequences (Fig. 5). Moreover, up to 10% of the OTUs, which accounts for 5% of the metazoan reads, had BLAST identities lower than 90%. The putative novelty was especially high among platyhelminthes, acoelomorphs, and nematodes, in which most of their OTUs (75%) had a BLAST identity lower than 97%. Gastrotrichs and crustaceans also had significant novelty (40–50% of their OTUs had a BLAST identity below 97%).

**Figure 6:**
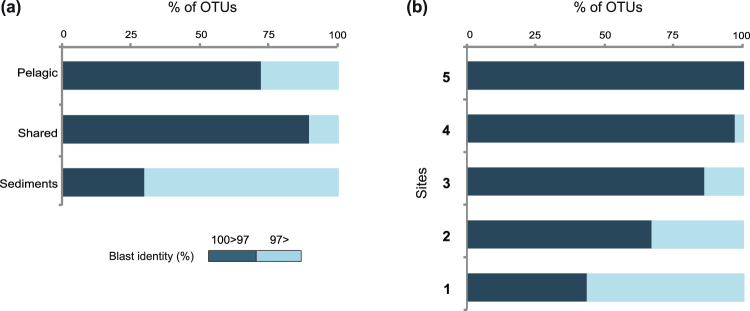
Sequence novelty regarding environmental and occurrence of different OTUs. Distribution of OTU BLAST identities against NCBI nt nr, in the OTUs classified by (**a**) environment (OTUs exclusively pelagic, benthc or present at both habitats) or (**b**) occurrence in different sites (OTUs present in one or up to 5 geographical sites).

Interestingly, the OTUs that appear to be most abundant within the water column (Table S5) and sediments (Table S6) correspond either to already known sequences or with high similarity to known sequences. The level of novelty is also different between benthic and pelagic environments. Thus, 70% of the OTUs found in benthic environments had a BLAST identity of less than 97% (Fig. 6a), while this percentage decreased to 21% of OTUs in the water column or to 11% of OTUs present in both water column and benthos. This suggests that benthic marine environments are a potential hot-spot to find new metazoan taxa or lineages.

Among the potential novelty, we detected a group of three OTUs with low levels of identity against our reference Metazoan database that had a relatively large proportion of RNA reads in oxic pelagic samples (1.8%) (Fig. 2a, labelled as “MAME 1”; MArine MEtazoan group 1). Interestingly, MAME 1 OTUs had more presence in micro/mesoplanktonic samples of Barcelona and Naples with 1–5% of metazoan RNA reads respectively. Although, we could not properly assign a taxonomy to those OTUs based on BLAST identities, we found two unclassified environmental sequences in GenBank (KC582969 and HQ869055) that were related with MAME 1 OTUs with identities around 95%. Those sequences came out to be useful in the further phylogenetic reconstruction of the group (see below and Methods). We failed to recover other sequences related to this group in SILVA or PR2 databases.

**Figure 7:**
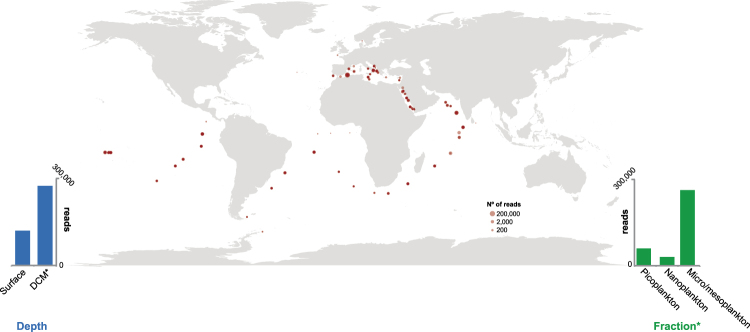
Worldwide distribution of MAME 1 group. World map within BioMarks data or within public repositories. Dot size represents the number of reads found shown on a log2 scale. Bar charts show the distribution of MAME 1 reads by depth and fraction.

Next, we decided to explore other HTES studies of 18S rDNA gene coming from marine pelagic samples. We found at SRA repository (https://www.ncbi.nlm.nih.gov/sra) 487 matching such characteristics. In 99 of them we found the presence of potential MAME 1 reads that were clustered in 14 different OTUs (see methods). In addition, we look at Tara Oceans^9^ data and we found other 52 OTUs that are potentially from the same MAME 1 clade. Those 69 OTUs from BioMarks, SRA and Tara Oceans represent 389,703 reads in total, an indication that OTUs assigned to this group are relatively common in marine environments. Indeed, we found that MAME 1 was present in coastal and open waters with a widespread distribution across the world’s oceans (except for the Arctic) in both the surface and the deep chlorophyll maximum (Fig. 7).

**Figure 8:**
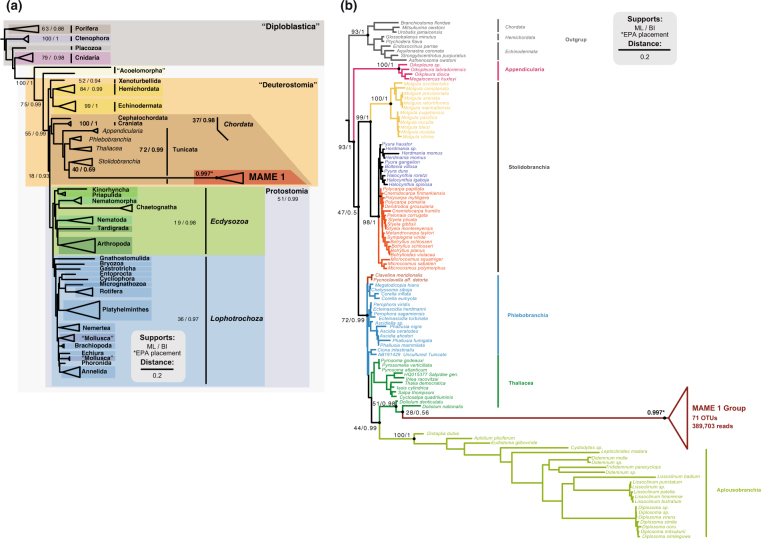
18S rRNA phylogenetic trees placing the novel metazoan group MAME 1. The trees were inferred using the NCBI long sequence *KC582969* as a representative of MAME 1 group. The nodal support values marked with a dot correspond to maximum likelihood 100-replicate bootstrap support and Bayesian posterior probabilities. The other 69 MAME 1 OTUs were placed to the group by the RAxML-EPA algorithm. The asterisk indicates that all MAME 1 OTU’s were placed with the MAME 1 sequence with an average likelihood on of 0.997 in both trees. In (**a**) the phylogenetic inference of MAME 1 within Metazoa. Metazoan super-group nomenclature is based on Paps *et al*. 2009a and b^66,67^. On the other hand (**b**) show its phylogenetic position within tunicates using the sequences from Tsagkogeorga *et al*.^62^.

To have a better understanding of its phylogenetic position, we performed phylogenetic trees. In a preliminary phylogenetic inference including only the three MAME 1 OTUs from our dataset and the longer sequences from NCBI sequences we found that they form a highly supported monophyletic clade (100 ML bootstrap and 1,0 Bayesian posterior probability). Thus, to place all the 69 MAME 1 OTUs, we decided to run a phylogenetic tree only including the longest NCBI sequence (KC582969) as a representative of the group, avoiding the problems of including many short sequences in the tree that would add a noisy signal. As a result, our trees placed the MAME 1 GenBank sequence within tunicates by both maximum likelihood and Bayesian inference (Table S3), and with good nodal support (79% bootstrap support and 0.99 Bayesian posterior probability), although with relatively longer branches than the rest of the metazoans (Fig. 8a). To determine its specific phylogenetic position within the tunicates, we inferred an additional tree with most of the available 18S rRNA sequences of tunicates, representing most of the known diversity of this phylum. In this tunicate-focused tree, the MAME 1 sequence clustered with thaliaceans as sister-group to the genus *Doliolium*, although with low nodal support (Fig. 8b). Finally, we ran a RAxML-EPA analysis to place the 69 OTUs plus the other NCBI sequence within the reference tree of metazoans and the tree of tunicates. In both cases, the 69 OTUs were placed with the reference MAME 1 sequences forming a monophyletic clade with a high likelihood weight. Most of the OTUs were placed to MAME 1 group with 1 likelihood weight, the maximum score. On average they were placed with a likelihood of 0.997 (Fig. 8). Thus, our phylogenetic analysis suggests that MAME 1 represents a monophyletic, novel and previously undescribed group of tunicates. Given their extremely long-branches, however, additional molecular data will be needed to further confirm this relationship.

## Discussion

### High-throughput sequencing, a powerful methodology to assess diversity

HTES is a useful methodology, but it also has some caveats. For example, it is well known that it may be misleading to directly translate reads and OTU numbers into biomass and number of species, respectively. In particular, the use of amplicon data as a proxy for metazoan biomass abundance has been disputed, also with RNA data^28^. Different number of rRNA copies in the genomes of different taxa, PCR primer mismatches and amplicon lengths can all affect the correlation between morphological and molecular data^7,29^. However, some studies have indeed shown positive correlations between read abundances and biomass patterns in bivalve and decapod larvae^23^, within copepod groups^7^ and specially using RNA data, which have been shown to be a better proxy for alpha-diversity^22^. Thus, we believe our approach to biomass abundance, although not perfect, is useful enough to report the most abundant groups. A good indication of our approach is that we recovered the general patterns previously described in micro/mesoplanktonic communities based on morphological observations^30,31^, in which copepods were found to be predominant within micro/mesoplanktonic communities^6^ followed by appendicularians^31^. Moreover, we found a more heterogenic distribution in benthic habitats, which is to be expected considering that sediments are known to harbor most of the metazoan diversity^5^. The use of RNA together with DNA data is not only important to infer alpha-diversity estimates, it also has permitted to have a wider view of the read distribution of metazoan OTUs, crucial in interpretation of, for instance, the presence of metazoan reads in the pico-nanoplanktonic samples. Additionally, allows a better recovery of the biological diversity (see rarefaction curves, Fig. S2).

Overall, our data confirms that, although with some caveats, HTES is a powerful tool to assess diversity. In this regard, the construction of a phylogenetically curated database to assign the OTU taxonomy has proven to be crucial for our analysis aimed at describing novelty in different metazoan phyla. Our clustering of OTUs at 97% is likely a conservative approach for metazoans^32^, and some of our OTUs may indeed represent more than one species. This largely depends on each metazoan lineage and its specific 18S rRNA evolution rate. Moreover, primer bias can affect the detection of some groups, meaning that some taxa can be present in the environment but missing in our dataset^33^. However, by clustering at 97% we can directly compare the results with the rest of the eukaryotes and get a more stringent output avoiding polymorphisms effects and an overrepresentation of the retrieved diversity.

### Benthic-Pelagic relationship

Analysis of benthic and pelagic metazoan communities in our dataset revealed that most OTUs are exclusively pelagic or benthic, showing few overlaps between the two communities, in agreement with our beta-diversity analyses (Figs S3, S4A) and the literature available^34,35^. Only 10% of OTUs from our dataset were present in both benthic and pelagic communities, and these mainly corresponded to polychaetes, crustaceans, molluscs and cnidarians (Fig. 3a). Among the shared OTUs Polychaeta and Mollusca water column reads probably represent juvenile pelagic stages^36,37^ while the benthic reads from crustaceans and cnidarians, that are predominantly pelagic, come likely from death organisms or debris.

In addition, our data clearly shows that the pelagic OTUs tend to be present in more sites (Fig. 3b), while most of the benthic OTUs are restricted to one location. The restricted presence of meiofaunal OTUs has been described previously^24^. Thus, the distribution in the water column fits more with the consideration that “everything is everywhere”^38^, probably because pelagic animals have fewer dispersal barriers than do benthic ones^39^.

### An ecological role for gametes?

Somewhat surprisingly, we observed a high percentage of metazoan reads in the smaller size fractions of most water column samples (Fig. 2). This includes, as well, the samples derived from RNA templates, probably indicating a significant biological activity of metazoans in those smaller fractions. We believe it is unlikely that those metazoan RNA reads could come from an extracellular origin because RNA is fragile and quickly degraded by ribonucleases, and its structure is easily affected by both oxygen and water^40^. Furthermore, the RNA reads from pico/nanoplanktonic fractions contain a different taxonomic distribution compared to the extracellular DNA samples and the micro/mesoplanktonic RNA samples (Figs 2a and 4a). Thus, and taking into account the small size reported for certain animal gametes, we hypothesize that a large part of those metazoan reads from the smaller fractions most likely come from metazoan gametes.

This is the case, for example, of the reads from smaller fractions assigned to tunicates, ctenophores, cnidarians and polychaetes, since they all use external fertilization. Ctenophora and Cnidaria, which are not only abundant in DNA reads but also have a relatively high number of RNA reads in the smaller fractions (Fig. 4b), might be a particularly notable example of the importance of gametes in the environment. The co-occurrence of reads in both smaller and larger fractions, the overrepresentation in the smaller ones and the fact that their sperm size is smaller than 5 µm^41,42^ are good indicators that at least the RNA signal of cnidarians and ctenophores might corresponds to gametes. That will not be the case for the reads assigned to copepods in the smaller fractions. They cannot come from gametes, since copepods use internal fertilization and release eggs larger than 50 µm^43^. Therefore, the crustacean RNA reads observed in smaller fractions (from 0.8 to 20 µm) are probably the result of cell breakage from larger fractions (Fig. 4a). Finally, we note that some of the OTUs that are exclusively retrieved from smaller fractions could also correspond to sperm from organisms that are larger than 2 mm or from benthic fauna with external fertilization and gamete sizes less than 10 µm, such as certain ctenophores and polychaetes (Table S7).

It is worth mentioning that metazoan RNA reads corresponding to germline cells could account, in our data, for as much as 3.2% of the total eukaryotic RNA reads in the smaller fractions (Table S7), and up to 33% of eukaryotic reads in anoxic samples. Thus, their numbers are comparable to those from the unicellular heterotrophic flagellates, which usually reach abundances of up to the 40% of eukaryotic RNA reads in pico and nano plankton^44^. Thus, and considering those abundances, sperm may play an important ecological role in those environments, particularly in the Black Sea anoxic waters. Further research is needed to assess the effect of sperm in microbial nutrient fluxes, especially during spawning events, when it may represent a passive member of the community eaten by other metazoans or protists from micro-scale fractions.

### Novelty in different metazoan phyla

We performed an analysis on novelty by plotting the pairwise identities of the first BLAST hit against NCBI non-redundant database. This provided a distribution of the “novel” OTUs (those with sequence identities lower than 97% to any NCBI sequence) along different environments (Fig. 6) and for different metazoan phyla (Fig. 5). Interestingly, we found that 45% of our metazoan OTUs had less than 97% identity against the NCBI nt nr database. Why a threshold of 97% for novelty? We believe it is the safest one to detect novelty, although we probably miss a lot of intra-genera or intra-class variation, depending in the animal group. It is worth mentioning, however, that by having a threshold of pair-wise identities below 97%, we avoid any potential intra-individual polymorphic variants^45^. Therefore, we follow the rationale that OTUs that do not have 100% identities but close (98% or higher) against the first BLAST hit from NCBI non-redundant database, are either the same taxa with different degrees of intraindividual variation or closely related species sharing the same genera/family. This depends on the evolution rate of 18S ribosomal gene across different metazoan groups. In contrast, the OTUs that have a BLAST identity under 97% represent much deeper changes, and so, they clearly represent, different taxa and most probably different families than the ones represented in GenBank. Some OTUs, especially those 10% of our OTUs with pairwise identities against GenBank under 90%, may even represent new clades.

Although one could argue that this degree of novelty might reflect sequencing artifacts, we are confident it is not the case because (1) we have followed a stringent chimera and singletons removal process, (2) the reads are distributed across different samples, and (3) they are not homogeneously distributed among taxonomic groups. In addition, around 80% of our OTUs have RNA reads and their taxonomic distribution is almost identical to the DNA OTUs. So, these novel variants present in the RNA subset are transcribed by active organisms and are less prone to be artifacts or rare variants^21^.

We are aware that detection of novelty in metazoans just with molecular data is challenging, given that the number of described animal species is larger than the number of 18S rRNA sequences available in public databases (Fig. S5B). Therefore, a novel sequence might belong to a species that has already been described but not yet sequenced. A complete database linking morphological and molecular data is needed to fully solve this issue. However, the 18S rRNA data so far available certainly is a good representation of known animal diversity (Fig. S5B), and we believe our study does indicates which metazoan lineages contain the higher levels of hidden molecular diversity, and so, which are the animal groups needed for a more extensive sampling.

Those animal groups with the higher levels of novelty are not others than crustaceans, nematodes, platyhelminthes, gastrotrichs and acoelomorphs. With the exception of crustaceans, these groups occupy early branching phylogenetic positions within the Ecdysozoa or the Lophotrochoa/Spiralia, or even within the Bilateria^46^. Moreover, the high genetic diversity in often neglected groups such as Acoelomorpha^17^ and Gastrotricha^10^ reveals that these groups need a deeper exploration.

We cannot rule out the possibility that the relatively fast evolutionary rates of the 18S sequences from nematodes, acoelomorphs and chaetognaths may have an effect on these low similarity values (identities below 90% against Genbank). In addition, intragenomic variability of the 18S rRNA gene, already described in some metazoan groups such as Platyhelminthes^47^ or Chaetognaths^48^, can also contribute to these novelty values. Nevertheless, those specific (intragenomic variability) or more common 18S rRNA variants (fast-evolving taxa), are a minor effect compared to the main issue of those groups with high novelty (nematodes, platyhelminthes, acoelomorphs, etc), which is the important unlink between all known morphological diversity and the molecular data available. A deeper exploration on those groups is needed if we want to fully understand metazoan diversity.

Thus, there is certainly extensive genetic novelty in our dataset, suggesting that most acoelomorph, platyhelminth, chaetognath, and nematode species have not yet been sequenced. Some of these hidden animal OTUs occupy key phylogenetic positions, which can help to better reconstruct the metazoan tree of life and unravel the evolution of extant species from the Urmetazoan^17^.

### A potential novel group of tunicates revealed by HTES

We also recovered and genetically described a potential novel group of tunicates, here named as “MAME 1”. It could be argued that this group represents an already described Thaliacean related to the genus *Doliolum* that happens to have never been sequenced or rare variants of the 18S gene belonging to known species. However, we consider these two options unlikely for several reasons. First, the group seems to be well populated (69 OTUs between our data and public repositories) and present in many environments worldwide, not only in coastal waters (Fig. S5). Moreover, the pairwise identity of the two MAME 1 sequences retrieved from NCBI is about 89%, suggesting is not a single species, but rather an entire group of sequences with high genetic variability, forming an independent clade related to Thaliaceans (Fig. 5). In fact, the nucleotide identity among MAME 1 OTUs is similar as the observed among distant *Aplousobranchia* species (for example, there is an 88% of identity between the 18S rRNA of *Distaplia dubia* and *Diplosoma virens*). Finally, different classes of the 18S rRNA gene have not yet been reported in Tunicates (there are 628 18S ribosomal tunicate sequences available at Genbank) and the percentage of identity of MAME 1 sequences against described Tunicate species seems too low (78% of identity with the best BLAST hit *Thalia democratica*) for a different 18S rRNA type. In animal groups in which different classes of 18S rRNA gene have been described, such as in chaetognaths, the intra-individual variation among 18S classes lies around 90–93% of identity^48^. Therefore, we suggest that MAME 1 might corresponds to a new group of tunicates that contains a large number of RNA reads within micro/mesoplankton environments and that is present in different habitats. However, without morphological data, we cannot truly discard the possibility that those sequences belong to a molecular divergent group of Thaliacean species, already morphologically described, but without genetic data available. Although this emphasizes the powerful of HTES to assess biodiversity and detect novelty, it also highlights its limitations. Thus, it is crucial to continue and improve the classical screenings of marine diversity, with the aim to link altogether morphological and genetic information in order to better understand the metazoan biodiversity of our oceans.

## Conclusions

We have reported an analysis of micrometazoan diversity in the European coast based on HTES that includes, for the first time, both water column and sediments, oxic and anoxic environments, and both DNA and RNA templates. To assess taxonomy, we constructed a novel reference dataset comprising all animal phyla, which was manually and phylogenetically curated. Our data show general read abundance and richness patterns that partially corroborate previous morphological^5,6,30,31^ and molecular studies^8,10,13,23,24,49^. Our data showed a high relative abundance of metazoan RNA reads within pico-nano size fractions (0.8–20 µm), suggesting that the sperm of ctenophores and cnidarians plays a relevant ecological role as part of the microbial food network. These results show the potential of HTES techniques as a fast and exhaustive method to approach the study of micrometazoan biomass and diversity patterns.

This kind of data has allowed us to describe novelty values found in different animal phyla. We observed that some animal phyla have much genetic novelty that is yet to be unraveled, including novelty in several well sampled groups such as Crustacea, Platyhelminthes or Nematoda. Our finding of a potential new group of widespread tunicates (MAME 1) highlights the value of phylogenetic approaches to identify novel groups within phyla. The finding of MAME 1 in several HTES datasets could be considered the first step in a reverse taxonomic process^50^ potentially leading to isolation and detailed description. Overall, our data show that, if we truly want to understand the biodiversity of marine environments, it is important to further sample animal taxa within those environments. To achieve that, we need to have better tools for the genetic screening, and especially for the isolation and morphological characterization of these organisms.

## Materials and Methods

### Sampling, 454 sequencing, curation of the sequences and OTU generation

During the BioMarKs project (biomarks.eu), samples were collected in six European coastal sites (Fig. S1; Table S1): the North Sea (Oslo, Norway), the English Channel (Roscoff, France), the Bay of Biscay (Gijón, Spain), the Mediterranean Sea (Blanes, Spain, and Naples, Italy) and the Black Sea (Varna, Bulgaria). Water column samples were taken with Niskin bottles attached to a CTD rosette at surface and deep chlorophyll maximum depths. Twenty liters of water per sample were pre-filtered through 20 μm filters and then sequentially filtered through 3 μm and 0.8 μm polycarbonate filters (diameter: 142 mm) using a peristaltic pump. Filtration time did not surpass 30 min to avoid RNA degradation. For dissolved DNA, 20 liters of 0.2 μm-filtered seawater was mixed with 400 ml of 0.5% CTAB (cetyltrimethylammonium bromide) (pH = 8) for 5 h and filtered through 0.2 μm polycarbonate membranes (142 mm). To collect the micro- (20–200 μm) and meso- (200–2000 μm) planktonic fractions (micro/mesoplankton), a plankton net of 20 μm mesh size was towed for 5–15 min, and the large protists collected were rinsed with 0.2 μm-filtered seawater, passed through a 2000 μm metallic sieve and filtered with 12 μm PC membranes (47 mm). Filters were flash frozen and stored at −80 °C. Sediment samples were taken with sediment cores with 11 cm depth and a diameter of 2.67 cm. Small aliquots of 10 ml were frozen at −80 °C (Table S1). The total number of samples considered in this study was 137 (Table S2). Total DNA and RNA were extracted at the same time from the same filter using the NucleoSpin RNA L kit (Macherey-Nagel, Düren, Germany).

The whole filter was used for DNA and RNA extractions in the pelagic samples. On the other hand, 5 g and 2 g of sediments were used for DNA and RNA extractions respectively. After quantification with a Nanodrop ND-1000 spectrophotometer (NanoDrop Technologies Inc, Wilmington, DE, USA), the quality was further checked on a 1.5% agarose gel. Contaminating DNA was removed from RNA samples using the TurboDNA kit (Ambion, Carlsbad, CA, USA). Extracted RNA was immediately reverse transcribed using the RT Superscript III random primers kit (Invitrogen, Carlsbad, CA, USA). The universal primers TAReuk454FWD1 (50-CCAGCASCYGCGGTAATTCC-30) and TAReukREV3 (50-ACTTTCGTTCTTGATYRA-30) were used to amplify the V4 region (~380 bp) of the eukaryotic 18S rDNA^45^. Primers were adapted for 454 following the manufacturer’s specifications. They had the configuration A-adapter-tag (7 or 8 bp)-forward primer and B-adapter-reverse primer.

PCRs were performed as explained in Logares *et al*.^44^, where amplifications were done in a volume of solution of 25 ml and consisted on a 1x MasterMix Phusion High-Fidelity DNA Polymerase (Finnzymes, Espoo, Finland), 0.35 mM of each primer and 3% DMSO. 5 ng of template DNA/cDNA was added to each PCR sample. PCRs cycles started with a denaturation step at 98 °C for 30 s, followed by 10 cycles of 10 s at 98 °C, 30 s at 53 °C and 30 s at 72 °C, and afterwards by 15 cycles of 10 s at 98 °C, 30 s at 48 °C and 30 s at 72 °C. The blanks used for the DNA/RNA extraction kit, and for PCR amplification, showed that samples were not contaminated with foreign DNA. Each PCR was repeated three times in order to have technical replicates. Triplicate amplicons were evaluated in a 1.5% agarose gel to check for successful amplifications and were pooled and purified using the NucleoSpin Extract II (Macherey-Nagel), eluted in 30 µl of elution buffer. The amplicons of each sample were quantified again using a Nanodrop ND-1000 spectrophotometer. At the end, we had pooled amplicons from 94 distinct samples coming both templates RNA and DNA (47 × 2 = 94), 9 DNA samples coming from extracellular material of pelagic samples (<0.2 µm) and 34 replicates of the 94 distinct samples (same RNA and DNA extraction, but different set of triplicated PCRs, with different 454 barcodes). All the amplicons pooled together represented approximately 5 µg of DNA and were sequenced with a 454 GS FLX Titanium system (454 Life Sciences, Branford, CT, USA) installed at Genoscope (http://ig.cea.fr/drf/ig/Pages/Genoscope.aspx, France). Pyroreads were inspected to remove short reads, reads with low quality and chimeras, as described in Massana *et al*.^18^ (Table S2).

Processed reads allowed to build a OTU (Operational Taxonomic Unit) table (reads per sample) with usearch v8.1.861^51^, using the UPARSE OTU clustering algorithm^52^, at a threshold of 97% similarity. Afterwards, we used our own metazoan reference dataset available at figshare (https://figshare.com/articles/Supplementary_Data_Lopez-Escardo_et_al_2016/3475007) to assign a taxonomical affiliation to our OTUs. Finally, we removed the putative chimeric metazoan sequences using Mothur’s Chimera Slayer^53^ and discarded all the singletons. We determined the degree of novelty of our dataset, by blasting the OTU sequences against NCBI nt nr (September 23 2014).

### Reproducibility of PCR and 454 sequencing

In order to evaluate the reproducibility of the different replicates, we selected duplicated samples from different templates (8 DNA, 9 RNA) and different sampling sites (2 Barcelona, 7 Naples and 8 Oslo), in which one duplicates contain at least more than 100 metazoan reads (n = 17). Each duplicate (same nucleic acid template and separate PCR and 454 adapters) was selected from our 34 duplicated samples out of 94 distinct samples. We calculated the linear regressions by plotting the OTU abundances in each duplicate^18^. The ratio between the total number of reads between both duplicates varied from 0.01 to 0.94. However, the regression line obtained by plotting all the OTU abundances in each duplicate showed that our duplicates were highly correlated, (Fig. S6). This means that the most abundant OTUs were systematically recovered in each duplicate and our molecular surveys were well suited for obtaining robust ß-diversity and taxonomic descriptions as described in Massana *et al*.^18^. The differences between the total number of reads among duplicates were due to replicates that have few number of total reads (<200). Luckily, most of the samples without replicates have high numbers of total reads, except few anoxic and pico-nanoplanktonic samples. Therefore we consider that our results show a reliable estimate of the metazoan diversity present in our samples.

### Diversity and distribution analysis

The metazoan OTU table obtained was processed for community analysis using QIIME v1.7.0^54^. Beta-diversity analyses including PCA and Jackknife clustering were performed with Unifrac^55^. The OTU tree used as input for Unifrac was constructed after aligning the OTUs with Mothur^53^. A subset of aligned sequences from our homemade database was used as a reference for Mothur input. Then, a maximum likelihood tree was generated with RAxML 7.2.8 and using GTRCATI as the evolutionary model. A hundred repeated runs on distinct starting trees were carried out to select the tree with the best topology and 100 bootstrap replicates were performed using the same evolutionary model. On the other hand, rarefaction curves were calculated under R environment^56^ using the Vegan v.2.3.0 library^57^.

Using QIIME scripts, we binned the OTUs that contain RNA reads within the water column of each sampling site into three different groups: 1) OTUs containing the small fractions (pico/nano), 2) OTUs containing the larger fraction (micro/meso), and 3) OTUs present in both small and large size classes. OTUs representing less than 10 RNA reads per site were discarded.

### Phylogenetic analysis of MAME1 sequence tags

In order to phylogenetically place the short reads assigned to the novel metazoan group (MAME 1; MArine MEtazoan group 1), we performed a RAxML-EPA analysis^58^. First, we built a metazoan reference tree using the longest putative MAME 1 sequence (1878 bp) found by BLAST at NCBI nt nr database (*KC582969*), as a unique MAME 1 representative. Metazoan 18S rRNA gene sequences were downloaded from GenBank (Table S3) and aligned using a MAFFT 7 E-INS-i strategy^59^. The resulting alignment was checked by eye with Geneious 8.0.4^60^, and the ambiguously aligned positions deleted, resulting in a total of 1472 nucleotide positions. Bayesian inference analysis was conducted with MrBayes 3.2.6^61^ using the GTR +Γ +invariant model of evolution running 6,000,000 generations. Maximum likelihood trees were generated with RAxML 7.2.8, using GTRCATI as the evolutionary model. To place the MAME 1 group within tunicates, an additional alignment was constructed with all tunicate sequences available and a phylogenetic tree was inferred using the same strategy. Tunicate sequences were mostly taken from Tsagkogeorga *et al*.^62^ who had an alignment of 110 sequences (95 from tunicates) occupying 1746 nucleotide positions. All the alignments and trees are available at figshare (https://figshare.com/articles/Supplementary_Data_Lopez-Escardo_et_al_2016/3475007).

To search for MAME 1 – like sequences in other metabarcoding studies we downloaded 487 marine pelagic environmental 18S amplicon datasets from NCBI’s SRA (March 2016) using fastq-dump from SRA-toolkit with -R option (List of amplicons available in the online supplementary material: https://figshare.com/articles/Supplementary_Data_Lopez-Escardo_et_al_2016/3475007), which selects the high quality reads^63^. We performed a BLAST search over the SRA dataset using KC582969 as a query and an e-value cut-off of e-100, retrieving 3677 putative MAME 1 reads from 104 SRA runs. Before processing them, we used PEAR^64^ to merge all the Illumina pair-end reads retrieved. Next, we used usearch v8.1.861 for quality filtering, dereplication, clustering (97%) and chimera checking using SILVA SSU 119^65^ as a reference. We ended up with 14 putative MAME 1 OTUs representing 3597 reads. We also performed a BLAST search (cut-off e value of e-10) against the Tara Oceans database^9^, retrieving 58 putative MAME 1 OTUs representing 123,779 reads.

We aligned all the MAME 1 – like short-read OTUs retrieved in the previous step and the ones from BioMarks with the representative sequences used for the metazoan and tunicate reference trees using the MAFFT strategy described earlier. After discarding sequences that did not align properly, we ended up with 69 MAME 1 OTUs (3 from BioMarKs, 14 from SRA and 52 from TARA), as well as the NCBI sequences KC582969 and *HQ869055*. Ambiguous positions were removed from the alignment checked by eye with Geneious 8.0.4^60^. The metazoan alignment yielded 1514 nucleotide positions, while the tunicate-specific alignment generated 1707 positions. Finally, we used RAxML-EPA to place the short reads in both the metazoan and the tunicate-specific datasets.

After the OTU assignments, we built an OTU table with the 69 MAME 1 group OTUs. We used QIIME to analyze their read abundance and distribution across different geographical locations, depths and size fractions. The OTU table and all the alignments and trees are available as supplementary information at figshare (https://figshare.com/articles/Supplementary_Data_Lopez-Escardo_et_al_2016/3475007).

### Data accessibility

Electronic supplementary material that accompanies the online version of this article includes materials and methods and supplementary figures and tables. The complete BioMarks sequencing dataset is available at European Nucleotide Archive (EMBL-EBI) http://www.ebi.ac.uk/ena, under project accession number PRJEB9133. OTU tables, 18S metazoan database, MAME 1 group OTU table and phylogenetic trees data (alignments, sequences and trees) are available at Figshare:https://figshare.com/articles/Supplementary_Data_Lopez-Escardo_et_al_2016/3475007.

## Electronic supplementary material

Supplementary Figure Legends and Tables
