# Supplementary material for: Metabarcoding analysis on European coastal samples reveals new molecular metazoan diversity
Source: Sci Rep. 2018 Jun 14;8:9106. doi: 10.1038/s41598-018-27509-8 (PMC6002407; doi:10.1038/s41598-018-27509-8)
Supplement: Supplementary file 1 — Supplementary Figure Legends and Tables [file 41598_2018_27509_MOESM1_ESM.pdf]

A research article submitted to Scientific Reports

Metabarcoding analysis on European coastal samples reveals new molecular metazoan diversity

David López-Escardó[1], Jordi Paps[2], Colomban de Vargas[3,4], Ramon Massana[5], Iñaki Ruiz-Trillo[1,6,7*], Javier del Campo[1,8*]

Supplementary Figure Legends and Tables

Fig. S1: Box plot distribution of relative metazoan abundance compared with all eukaryotes. Relative abundance of metazoans compared to all eukaryotes in **(a)** different oxic pelagic fractions, **(b)** different sites and in (**c**) different depths. Note that data is provided from just one sample in the anoxic sediments.

Fig. S2: Rarefaction curves. Rarefaction curves calculated with vegan from the samples divided **(a)** by template (RNA or DNA) or **(b)** by environment keepig as well divided the samples from RNA (discontinuous line) or DNA (continuous line). Both plots show the rarefaction curve of all the samples.

Fig. S3: Jackknife clustering analysis of phylogenetic composition of the samples. The chart represents the relative abundance within metazoan phyla in each sample. Samples from extracellular DNA and the ones with less than 100 reads (DNA+RNA) were removed from the analysis. Sample characteristics are expressed in colors. On the right site there is the legend. RNA and DNA are expressed in white and black respectively. Picoplanktonic, nanoplanktonic, micromesoplanktonic samples and the samples from the sediments are representedwith yellow, green, dark red and purple respectively. Finally the samples from Oslo, Naples, Barcelona, Gijón, Roscoff and Varna were represented by green, cyan, red, orange, pink and purple respectively.

Fig. S4: Principal component analysis of the samples. Samples from extracellular DNA and the ones with less than 100 reads (DNA+RNA) were removed from this analysis. Analyses are shown for **(a)** size fraction, **(b)** depth and **(c)** site.

Fig. S5: Comparison of number of OTUs found, number of described metazoan species and number of 18S rRNA metazoan sequences in NCBI. (**a**) Total number of OTUs from 18S rRNA retrieved in our dataset (blue bars) compared to the number of described species for each metazoan phylum (red bars). (**b**) Total number of OTUs from 18S rRNA retrieved from Genbank (blue bars) compared to the number of described species for each metazoan phylum (red bars). The number of 18S rRNA sequences from NCBI was obtained from the following search for each phylum: "*txid33208[Organism:exp] (18S OR SSU) NOT (mitochondrial OR mitochondria)*". (B) MAME 1 distribution.

Fig. S6: Linear regression statistics of the read distribution between the duplicated samples analysed (n=17). The figure shows on the Y axis R^2 coefficients (blue dots) and slope values (read dots) of the regression line obtained by ploting all the OTU abundances in each duplicate. On the X axis it is showed the ratio of total number of reads between both duplicates. Note that even it was a variation between the total number of reads between the duplicates (pyrotag ratio between 0.01-0.94), this number correlates with the slope of the regression line and duplicated pairs were usually very similar in relative abundances (range of R^2 from 0.8 to 0.99, independent of the ratio of total number of reads between each duplicate), systematically retrieving the same dominant OTUs.

Table S1. Description of the sampling sites.

Site	Coordinates	Distance to coast (Km)	Max. Depth (m)[1]	Sampling date	DCM (m)	Temperature Surface (ºC)	Temperature DCM (ºC)	Temperature Sediment (ºC)	Salinity Surface (PSU)	Salinity Sediment (PSU)	Chla (µg/l)[2]	[NO_3^-] Surface/ DCM (µg/l)	[PO_4^{3-}] Surface/ DCM (µg/l)	Total Phosphorus Surface/DCM (µg/l)
**Blanes**	41° 40' N 2° 48' E	1.0	20	2/2010	N/A	12.5	N/A	12.6	37.5	38.2	1.0	2 / N/A	7 / N/A	13 / N/A
**Gijon**	43° 40' N 5° 35' W	12.0	110	9/2010	40	20.2	14.0	12.0	35.7	36.6	7.0	2 / 26	3 / 4	10 / 12
**Naples 2009**	40° 48' N 14° 15' E	4.0	75	10/2010	23	22.8	22.4	14.6	37.7	37.9	1.4	16 / 0	1 / 1	22 /16
**Naples 2010**	40° 48' N 14° 15' E	4.0	76	5/2010	35	19.2	15.5	14.0	37.2	37.9	1.2	<2 / <2	4 / 3	14 / 8
**Oslo 2009**	59° 16' N 10° 43' E	1.5	100	09/2010	8	15.0	15.0	8.0	25.0	35.0	3.2	9 / 1	4 / 3	22 / 21
**Oslo 2010**	59° 16' N 10° 43' E	1.5	100	06/2010	9	15.0	12.5	6.0	22.0	35.0	1.9	<2 / <2	3 / 2	12 / 11
**Roscoff**	48° 46' N 3° 57' W	5.0	60	4/2010	N/D	9.9	N/D	9.9	34.9	34.9	0.5	87 / N/D	12 / N/D	29 / (N/D)
**Varna**	43°10' N 28° 50' E	40.0	400	5/2010	40	21.5	9.5	8.5	16.0	22.0	8.0	2 / 2	4 / 3	11 / 11

Surface is considered as < 5 m depth. N/A= not applicable, N/D= no data.

[1] Maximum depth of the water column.

[2] Maximum Chlorophyll a concentration in the water column measured with fluorometry (fluorometer attached to a CTD).

Table S2 Summary of samples including the total number of eukaryotic reads after quality control and the total number of metazoan reads after all the filtering process and the OTU assignment.

	All eukaryotes				Metazoans			
**Description**	**Samples**	**Total reads**	**DNA reads**	**RNA reads**	**Samples**	**Total reads**	**DNA reads**	**RNA reads**
Site								
Blanes	11	94366	48813	45553	11	59626	35719	23907
Gijon	4	50178	29116	21062	4	233	199	34
Naples	46	600756	266549	334207	44	131168	70170	60998
Oslo	44	406563	224876	181687	44	93635	60637	32998
Roscoff	9	55567	41861	13706	9	17127	14731	2396
Varna	25	250977	122751	132006	25	71145	39550	31595
Size Fraction (µm)								
0-0.2 (eDNA)	8	44564	44564	n/a	8	7879	7879	n/a
0.8-3	38	439410	208067	231343	38	37575	22981	14594
3-20	36	394879	194915	199964	36	23570	15949	7621
20-2000	33	379910	187405	192505	31	222149	127190	94959
Depth								
Subsurface	63	624138	336875	287263	23	81761	46996	34765
DCM	45	536884	262120	274764	62	134470	85545	48925
Anoxic	7	101004	37328	63676	45	105932	63027	42905
Sediments	24	216013	111294	104409	7	50771	25438	25333
Template								
DNA	74	746245	n/a	n/a	72	220766	n/a	n/a
RNA	65	728221	n/a	n/a	65	152168	n/a	n/a
Total	n/a	1474466	746245	728221	n/a	3792934	220766	152168

Table S3. Summary of the sequences from GenBank used to place new MAME 1 group within metazoans in Figure 5. The summary includes the accession number of the 18S rRNA gene sequence, the specie that it belongs and its taxonomy.

Taxonomy	Specie	Accesion Number
**Porifera**	*Leucosolenia sp.*	AJ622898
	Oscarella carmela	EU702422
	Aphrocallistes beatrix	FM946127
	Petrosia sp.	DQ927321
**Ctenophora**	*Mertensia ovum*	AF293679
	Pleurobrachia bachei	AF293677
	Mnemiopsis leidyi	AF293700
**Placozoa**	*Trichoplax sp.*	Z22783
**Cnidaria**	*Aurelia aurita*	AY039208
	Coryne pusilla	Z86107
	Alcyonium gracillimum	Z92902
	Parazoanthus axinellae	U42453
	Nematostella vectensis	AF254382
	Actinia equina	AJ133552
	Anemonia sulcata	X53498
**Acoelomorpha**	*Paratomella rubra*	AF102892
	Nemertoderma westbladi	AF327726
**Xenoturbellida**	*Xenoturbella bocki*	AY291292
**Hemichordata**	*Saccoglossus pusillus*	AF236800
	Balanoglossus carnosus	D14359
	Glossobalanus minutus	AF119089
	Ptychodera flava	AF278681
**Echinodermata**	*Endoxocrinus parrae*	Z80951
	Antedon serrata	D14357
	Strongylocentrotus purpuratus	L28055
	Asthenosoma owstoni	Z37118
	Aquilonastra coronata	AB084566
	Astropecten latespinosus	AB084546
**Cephalochordata**	*Branchiostoma floridae*	M97571
**Craniata**	*Urobatis jamaicensis*	AY049861
	Mitsukurina owstoni	AY049840
**Tunicata**	*Oikopleura sp.*	AB013015
	Oikopleura labradoriensis	FM244869
	Ascidia sydneiensis	AF165819
	Ciona intestinalis	AB013017
	Clavelina meridionalis	FM244840
	Pyrostremma spinosum	HQ015379
	Thalia sp.	AB859895
	Salpidae sp.	HQ015377
	Halocynthia igaboja	AY903925
	Cnemidocarpa humilis	FM244859

	Molgula occidentalis	FM244850
	Molgula provisionalis	L12434.2
**MAME 1**	*Uncultured Eukaryote*	KC582969
	Uncultured Eukaryote	HQ869055
**Kinorhyncha**	*Pycnophyes kielensis*	U67997
**Priapullida**	*Halicryptus spinulosus*	AF342790
	Priapulus caudatus	Z38009
**Nematomorpha**	*Gordius albopunctatus*	U88337
	Neochordodes occidentalis	AF421768
	Paragordius tricuspidatus	AF421771
**Chaetognatha**	*Eukrohnia bathypelagica*	DQ351896
	Parasagitta setosa	DQ351900
	Sagitta bipunctata	DQ351890
**Nematoda**	*Enoplus brevis*	U88336
	Desmodora ovigera	Y16913
	Catanema sp.	Y16912
**Tardigrada**	*Milnesium tardigradum*	U49909
**Arthropoda**	*Colossendeis sp.*	AF005440
	Pandinus imperator	AY210831
	Limulus polyphemus	L81949
	Lithobius obscurus	AF334271
	Orthoporus sp.	AY210829
	Heterothrips arisaemae	KC512970
	Ctenolepisma longicaudata	AY210811
	Triops longicaudatus	AF144219
	Orchesellides sinensis	KC236251
	Squilla empusa	L81946
**Gnathostomulida**	*Haplognathia simplex*	DQ079931
**Bryozoa**	*Frondipora verrucosa*	FJ409612
**Gastrotricha**	*Paraturbanella teissieri*	JF357661
**Entoprocta**	*Barentsia benedeni*	U36272
**Cycliophora**	*Symbion pandora*	AY218106
**Micrognathozoa**	*Limnognathia maerski*	AJ487046
**Rotifera**	*Philodina roseola*	AF154567
	Brachionus plicatilis	U49911
	Lecane bulla	AF154566
	Asplanchna sieboldi	AF092434
**Platyhelminthes**	*Catenula sp.*	AJ012532
	Stenostomum leucops	D85095
	Macrostomum hystricinum	AF051329
	Haplopharynx rostratus	AJ012511
	Pseudoceros tritriatus	AJ228794
	Thysanozoon brocchii	D85096
	Planocera multitentaculata	D83383

	Discocelis tigrina	U70078
	Notoplana australis	D85097
**Nemertea**	*Amphiporus ochraceus*	AY039668
	Cerebratulus lacteus	AY145368
	Lineus ruber	AY039672
**Mollusca**	*Rhabdus rectius*	AF120523
	Lima lima	AF120533
	Pteria hirundo	AF120532
	Nuculana minuta	DQ279938
	Yoldia myalis	AF207643
**Brachiopoda**	*Glottidia palmeri*	U12647
	Lingula anatina	X81631
	Phoronis australis	U36271
**Annelida**	*Urechis caupo*	AF342805
	Aspidosiphon misakie	AF119090
	Phascolosoma granula	X79874
	Dero digitata	DQ459984
	Acanthobdella peledin	AY040680
	Glossiphonia complana	AF099943
	Erpobdella octoculata	AF099949

Table S4. Summary of reads and OTUs obtained within all metazoan phyla in BioMarks data.

Metazoan Phyla	OTUs	Reads	Metazoan Phyla	OTUs	Reads
**Porifera**	13	262	**Priapulida**	2	117
**Ctenophora**	10	**23,371**	**Kinorhyncha**	3	2,291
**Cnidaria**	**55**	**18,627**	**Nematoda**	**247**	**8,932**
**Acoelomorpha**	**31**	3,069	**Tardigrada**	3	8
**Gastrotricha**	**23**	1,540	**Arthropoda**		
**Gnathostomulida**	1	33	Myriapoda	1	190
**Rotifera**	12	**3,576**	Hexapoda	3	12
**Bryozoa**	12	1,055	Crustacea	**370**	**190,872**
**Phoronida**	1	306	Chellicerata	7	575
**Platyhelminthes**	**33**	1,540	**Chaetognatha**	**21**	**14,739**
**Nemertea**	7	622	**Xenoturbellida**	1	15
**Mollusca**	**53**	**9,907**	**Echinodermata**	16	3,206
**Annelida**			**Hemichordata**	2	123
Polychaeta	**97**	**34,693**	**Craniata**	14	1,961
Clitellata	1	5	**Tunicata**	**30**	**37,982**
Sipuncula	3	681	**MAME 1**	3	1,860

Table S5. Summary of the 20 most abundant metazoan OTUs in the water column taking into account the number of RNA reads.

Metazoan group	OTU	RNA reads	%[a] within metaz.	%[b] within group	CIM[c]	CIM BLAST % ID[d]	CIM taxonomy
**Crustacea**	6	10746	9.8%	22.0%	HM997070	100%	*Paracalanus parvus* (Calanoida, Copepod)
	2	7944	7.3%	16.3%	JX995318	100%	*Calanus helgolandicus* (Calanoida, Copepod)
	21	4834	4.4%	9.9%	JX995321	100%	*Pseudocalanus elongatus* (Calanoida, Copepod)
	11	3545	3.3%	7.3%	JX995298	100%	*Centropages typicus* (Calanoida, Copepod)
	15	3093	2.8%	6.3%	HM997062	100%	*Temora discaudata* (Calanoida, Copepod)
	84	2796	2.6%	5.7%	HM997079	98%	*Clausocalanus arcuicornis* (Calanoida, Copepod)
	224	2679	2.5%	5.5%	HM997079	99%	*Clausocalanus arcuicornis* (Calanoida, Copepod)
	22	2219	2.0%	4.5%	JF781540	98%	*Oithona sp.* (Cyclopoida, Copepod)
	8	1865	1.7%	3.8%	GU969179	100%	*Oithona Similis* (Cyclopoida, Copepod)
Total		39721	36.4%	81.2%			
**Tunicata**	4	21436	19.6%	56.6%	AB013014	100%	*Oikopleura dioica* (Appendicularian)
	39	1723	1.6%	4.6%	AY116613	100%	*Oikopleura dioica* (Appendicularian)
	169	1082	1.0%	2.9%	AB013012	100%	*Doliolum nationalis* (Doliolid)
Total		24241	22.2%	64.0%			
**Ctenophora**	7	12031	11.0%	83.5%	AF100944	100%	*Pleurobrachia pileus* (Typhlocoela)
	52	2108	1.9%	14.6%	AF293700	100%	*Lampocteis cruentiventer* (Cyclocoela)
Total		22387	12.9%	98.2%			
**Chaetognatha**	49	3736	3.4%	42.3%	DQ351879	99%	*Krohnitta pacifica* (Saggitoidea)
	80	3285	3.0%	37.2%	DQ351877	99%	*Flaccisagitta enflata* (Saggitoidea)
Total		7021	6.4%	79.5%			
**Cnidaria**	79	2114	1.9%	46.3%	AY039208	100%	*Aurelia aurita* (Scyphozoa)
	50	1887	1.7%	41.3%	DQ080014	100%	*Lilyopsis rosea* (Hydrozoa)
Total		4001	3.7%	87.6%			
**Rotifera**	116	2573	2.4%	86.9%	DQ297711	99%	*Notommata cordonella* (Ploimida)
**MAME 1**	102	1093	1.0%	65.1%	HQ869055*	95%	*Uncultured eukaryote**

a. Percentage of RNA reads within the metazoans in the water column.
b. Percentage of RNA reads within the correspondent in the water column.
c. Accession number of the Closest Identified Match.
d. BLAST identity.
* The Closest Identified Match match has a very low identity (less than 90%). It is indicated instead, the Closest Environmental Match (CEM).

Table S6. Summary of the 20 most abundant metazoan OTUs in the sediments taking into account the number of RNA reads.

Metazoan group	OTU	RNA reads	%[a] within metaz.	%[b] within group	CIM[c]	CIM BLAST % ID[d]	CIM taxonomy
**Polychaeta**	61	1823	5.2%	22.7%	EU340097	99%	*Aurospio foodbancsia* (Spionida)
	89	1742	5.0%	21.7%	JF903633	100%	*Prosphaerosyllis magnoculata* (Aciculata)
	88	1423	4.1%	17.7%	JN936464	92%	*Paralysippe annectens* (Scolecida)
	26	643	1.9%	8.0%	AF412798	99%	*Parougia sp.* (Aciculata)
	36	495	1.4%	6.2%	JN936464	92%	*Paralysippe annectens* (Scolecida)
	69	389	1.1%	4.8%	AY838852	100%	*Ninoe nigripes* (Aciculata)
	229	388	1.1%	4.8%	AY611455	100%	*Polydora giardi* (Scolecida)
Total		6903	19.8%	85.8%			
**Crustacea**	68	2900	8.3%	36.7%	AB076635	98%	*Limnocythere sp.* (Ostracoda)
	211	802	2.3%	10.2%	AB076621	89%	*Kotoracythere inconspicua* (Ostracoda)
	273	458	1.3%	5.8%	EU380309	97%	*Itunella muelleri* (Copepoda)
	216	458	1.3%	5.8%	AY627016	100%	*Brayda sp.* (Copepoda)
	76	399	1.2%	5.1%	AB076631	100%	*Leptocythere lacertosa* (Ostracoda)
	260	326	0.9%	4.1%	EU380293	97%	*Paramphiascella fulvofasciata* (Copepoda)
Total		5343	15.3%	67.6%			
**Mollusca**	18	5389	15.5%	82.9%	DQ279940	100%	*Abra nitida* (Bivalvia)
	67	847	2.4%	13.0%	EF489348	100%	*Scaphander lignarius* (Gastropoda)
Total		6236	17.9%	95.9%			
**Platyhelminthes**	74	1427	4.1%	48.3%	FJ715296	99%	*Microstomum papillosum* (Rhabditophora)
**Echinodermata**	51	1159	3.3%	92.8%	AJ011142	99%	*Amphiura chiajei* (Ophiurida)
**Nematoda**	176	624	1.8%	12.5%	AJ966473	94%	*Anaplectus sp.* (Chromadorea)
**Sipuncula**	340	500	1.4%	91.2%	AF519248	100%	*Phascolion strombus* (Golfingiida)
**Cnidaria**	50	337	1.0%	67.4%	DQ080014	100%	*Lilyopsis rosea* (Hydrozoa)

a. Percentage of RNA reads within the metazoans in the sediments.
b. Percentage of RNA reads within the correspondent in the sediments.
c. Accession number of the Closest Identified Match.
d. BLAST identity.

Table S7. Summary of the OTUs, whose RNA reads from small fractions are suspected to come from gametes.

Distribution	Metazoan group	OTU	Reads[c] within small fraction	%[d] within dataset	CIM	CIM BLAST % ID.	CIM taxonomy
**Small and large fractions[a]**	Ctenophora	7	11201	50.3%	AF100944	100%	*Pleurobrachia pileus* (Typhlocoela)
	Cnidaria	79	1589	7.1%	AY039208	100%	*Aurelia aurita* (Scyphozoa)
	Total		**12790**	**57.5%**			
**Small exclusive[b]**	Polychaeta	88	340	1.5%	JN936464	92%	*Paralysippe annectens* (Scolecida)
	Ctenophora	52	212	1.0%	KJ754158	100%	*Mnemiopsis leidy* (Cyclocoela)
	Ctenophora	5798	104	0.5%	KJ754154	98%	*Pleurobrachia brunnea* (Typhlocoela)
	Ctenophora	715	77	0.4%	HG931678	96%	*Beroe ovata* (Cyclocoela)
	Polychaeta	19	70	0.3%	AY611452	88%	*Hydroides novegica* (Palpata)
	Total		**803**	**3.6%**			
TOTAL[e]			**13593**	**3.2%[e]**			

a. OTUs that are present in pico/nano and micro/meso fractions.
b. The 5 most abundant OTUs exclusive from the pico/nano fractions.
c. Number of RNA reads within the pico/nano fractions.
d. Percentage of RNA reads within the pico/nano fractions for metazoans.
e. Percentage of RNA reads within the pico/nano fractions for all eukaryotes.

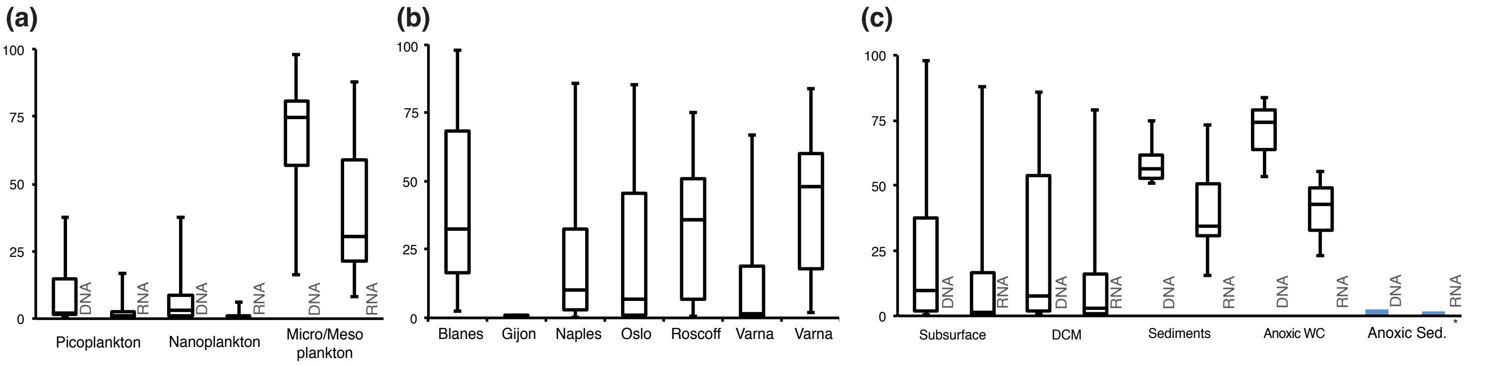
(a)
100
75
50
25
0
DNA
RNA
Picoplankton
Nanoplankton
Micro/Meso plankton
(b)
Blanes
Gijon
Naples
Oslo
Roscoff
Varna
Varna
(c)
Subsurface
DCM
Sediments
Anoxic WC
Anoxic Sed.
*

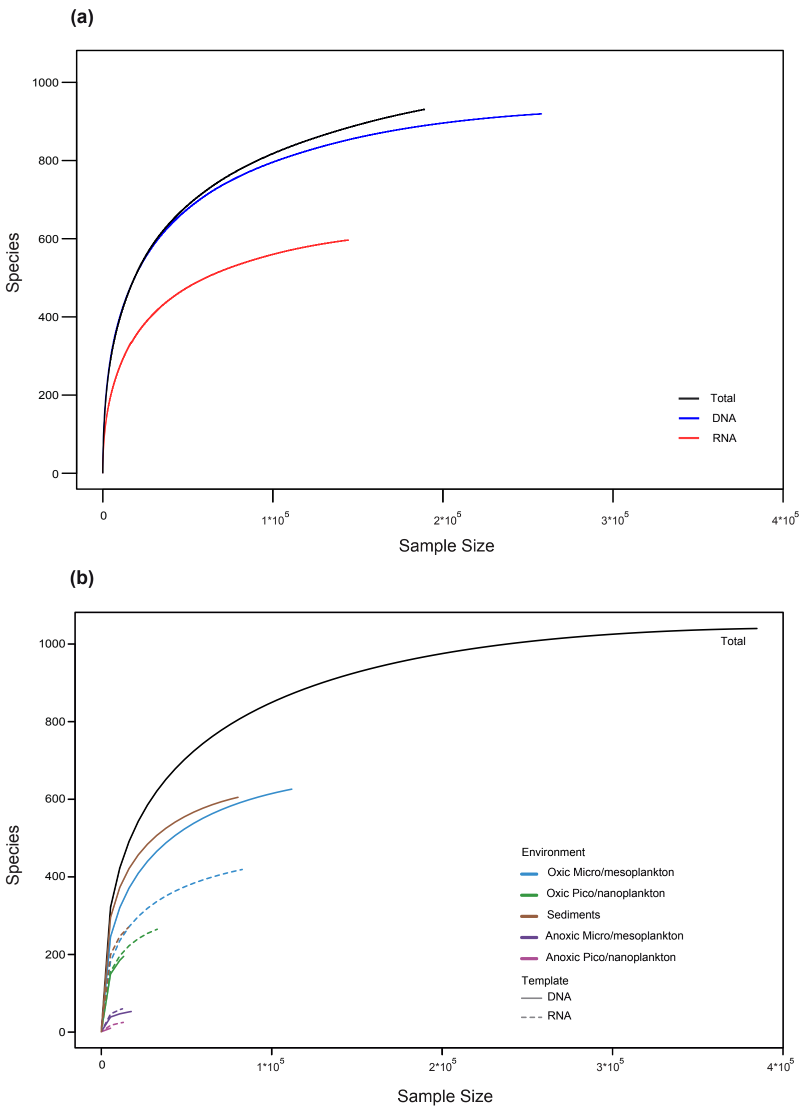
(a)
1000
800
600
400
200
0
Species
0
1*10^5
2*10^5
3*10^5
4*10^5
Sample Size
Total
DNA
RNA
(b)
1000
800
600
400
200
0
Species
0
1*10^5
2*10^5
3*10^5
4*10^5
Sample Size
Total
Environment
Oxic Micro/mesoplankton
Oxic Pico/nanoplankton
Sediments
Anoxic Micro/mesoplankton
Anoxic Pico/nanoplankton
Template
DNA
RNA

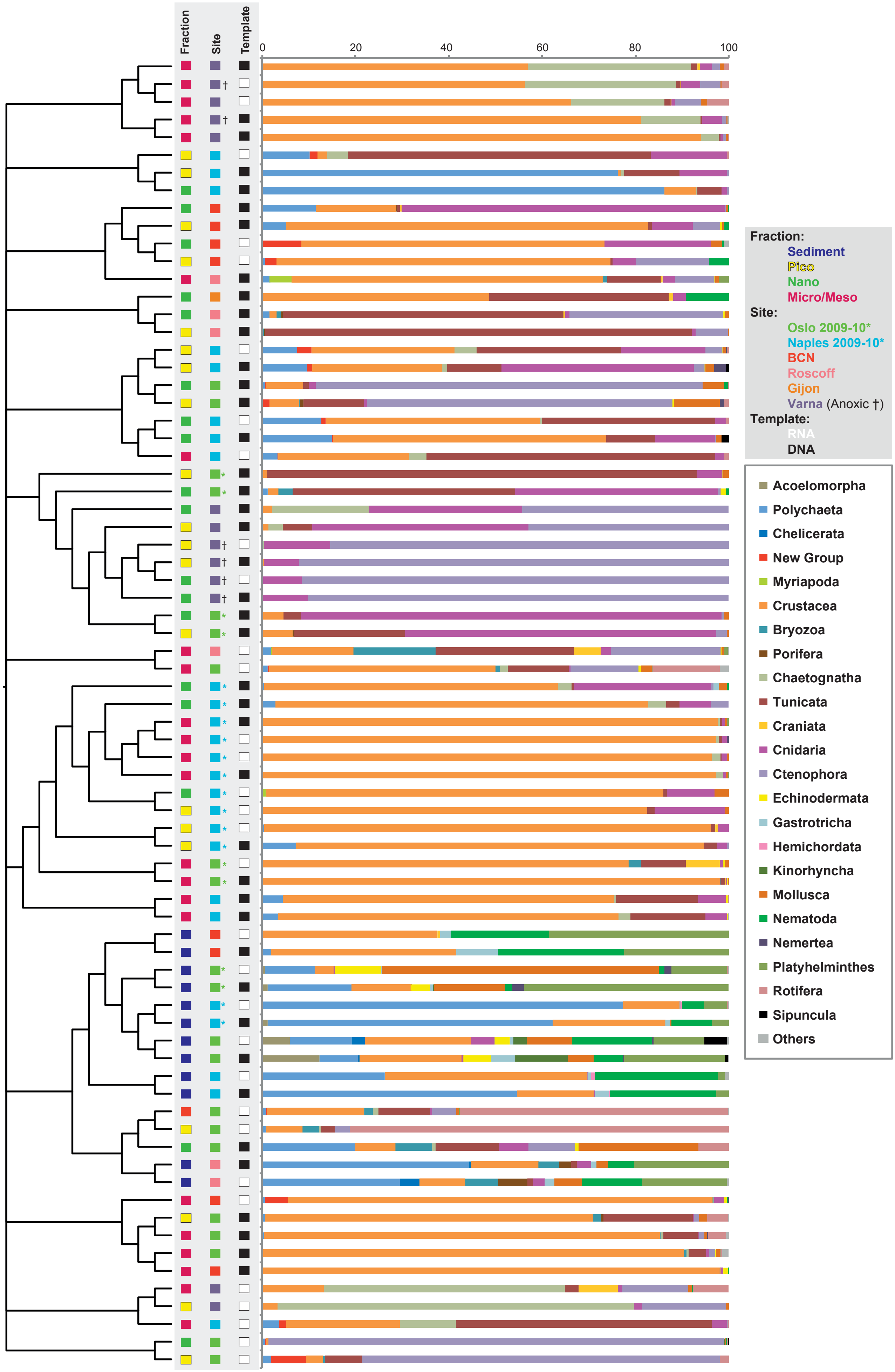
Fraction
Site
Template
0
20
40
60
80
100
Fraction:
Sediment
Pico
Nano
Micro/Meso
Site:
Oslo 2009-10*
Naples 2009-10*
BCN
Roscoff
Gijon
Varna (Anoxic †)
Template:
RNA
DNA
Acoelomorpha
Polychaeta
Chelicerata
New Group
Myriapoda
Crustacea
Bryozoa
Porifera
Chaetognatha
Tunicata
Craniata
Cnidaria
Ctenophora
Echinodermata
Gastrotricha
Hemichordata
Kinorhyncha
Mollusca
Nematoda
Nemertea
Platyhelminthes
Rotifera
Sipuncula
Others

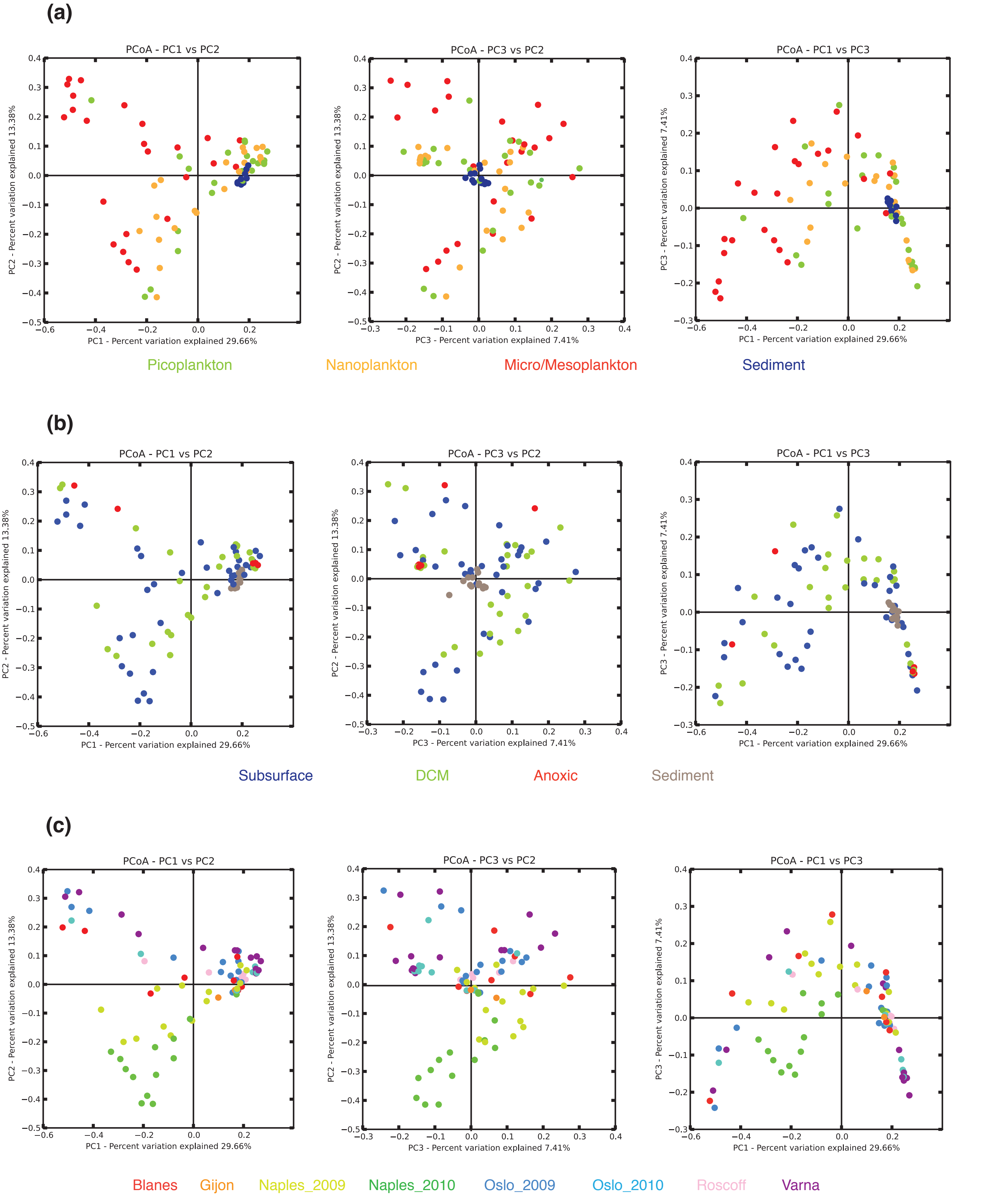
(a)
PCoA - PC1 vs PC2
PCoA - PC3 vs PC2
PCoA - PC1 vs PC3
PC1 - Percent variation explained 29.66%
PC2 - Percent variation explained 13.38%
PC3 - Percent variation explained 7.41%
Picoplankton
Nanoplankton
Micro/Mesoplankton
Sediment
(b)
PCoA - PC1 vs PC2
PCoA - PC3 vs PC2
PCoA - PC1 vs PC3
PC1 - Percent variation explained 29.66%
PC2 - Percent variation explained 13.38%
PC3 - Percent variation explained 7.41%
Subsurface
DCM
Anoxic
Sediment
(c)
PCoA - PC1 vs PC2
PCoA - PC3 vs PC2
PCoA - PC1 vs PC3
PC1 - Percent variation explained 29.66%
PC2 - Percent variation explained 13.38%
PC3 - Percent variation explained 7.41%
Blanes
Gijon
Naples_2009
Naples_2010
Oslo_2009
Oslo_2010
Roscoff
Varna

(a)

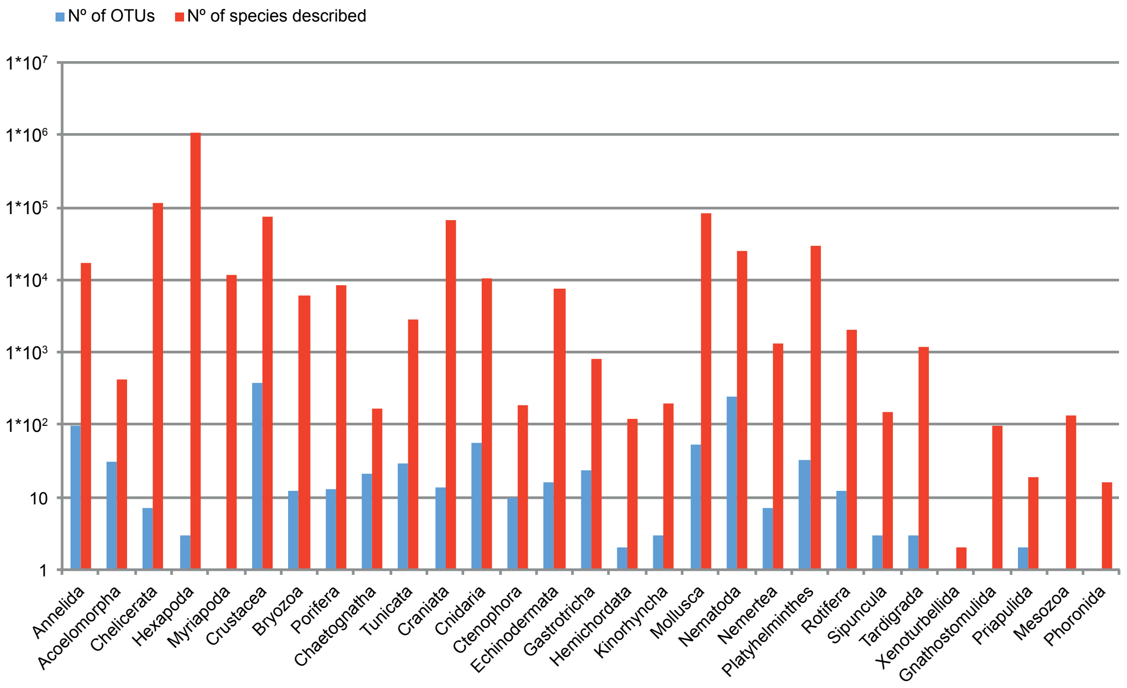

Nº of OTUs
Nº of species described
1*10^7
1*10^6
1*10^5
1*10^4
1*10^3
1*10^2
10
1
Annelida
Acoelomorpha
Chelicerata
Hexapoda
Myriapoda
Crustacea
Bryozoa
Porifera
Chaetognatha
Tunicata
Craniata
Cnidaria
Ctenophora
Echinodermata
Gastrotricha
Hemichordata
Kinorhyncha
Mollusca
Nematoda
Nemertea
Platyhelminthes
Rotifera
Sipuncula
Tardigrada
Xenoturbellida
Gnathostomulida
Priapulida
Mesozoa
Phoronida

(b)

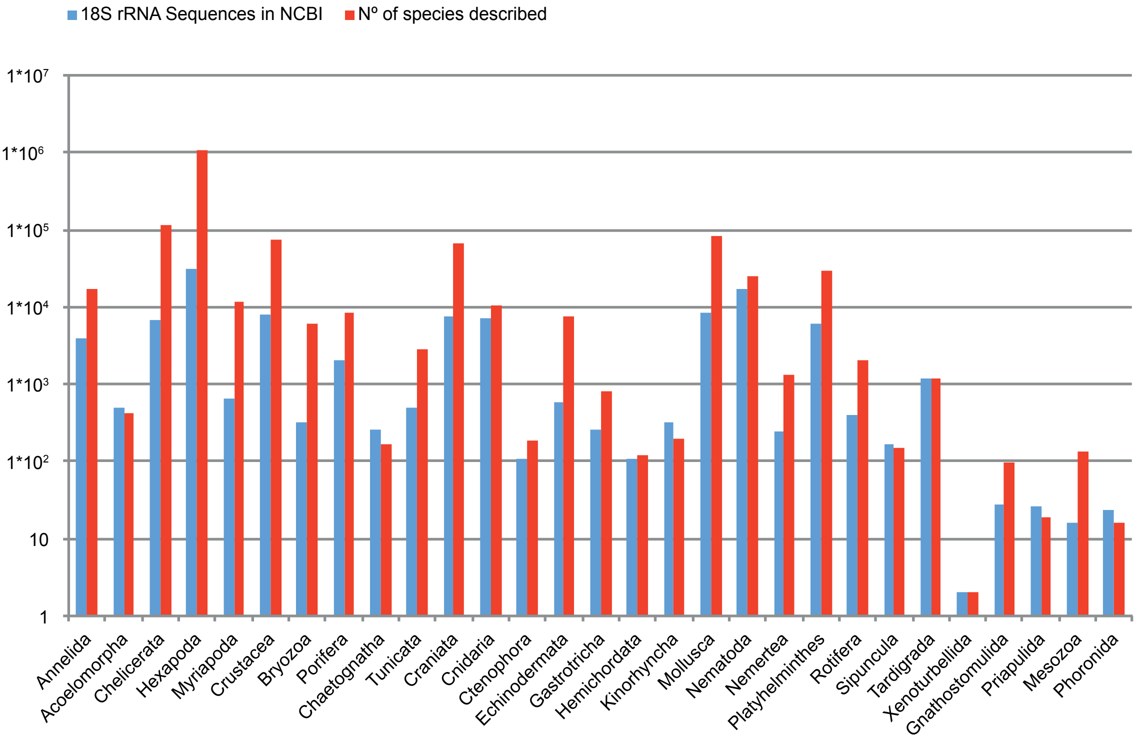

18S rRNA Sequences in NCBI
Nº of species described
1*10^7
1*10^6
1*10^5
1*10^4
1*10^3
1*10^2
10
1
Annelida
Acoelomorpha
Chelicerata
Hexapoda
Myriapoda
Crustacea
Bryozoa
Porifera
Chaetognatha
Tunicata
Craniata
Cnidaria
Ctenophora
Echinodermata
Gastrotricha
Hemichordata
Kinorhyncha
Mollusca
Nematoda
Nemertea
Platyhelminthes
Rotifera
Sipuncula
Tardigrada
Xenoturbellida
Gnathostomulida
Priapulida
Mesozoa
Phoronida

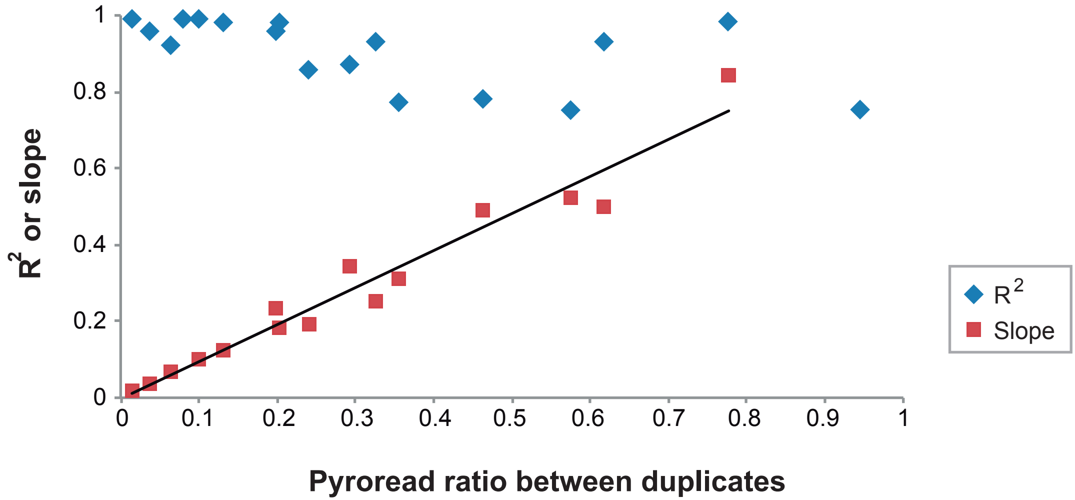

R^2 or slope
Pyroread ratio between duplicates
R^2
Slope
0
0.2
0.4
0.6
0.8
1
0
0.1
0.2
0.3
0.4
0.5
0.6
0.7
0.8
0.9
1
